# Contextualising the pervasive impact of macroeconomic austerity on prison health in England: a qualitative study among international policymakers

**DOI:** 10.1186/s12889-019-7396-7

**Published:** 2019-08-05

**Authors:** Nasrul Ismail

**Affiliations:** Centre for Public Health & Wellbeing, University of the West of England (UWE Bristol), Frenchay Campus, Coldharbour Lane, Bristol, BS16 1QY United Kingdom

**Keywords:** Prison, incarceration, austerity, neoliberalism, England

## Abstract

**Background:**

Prisons offer the state the opportunity to gain access to a population that is at particularly high risk of ill-health. Despite the supportive legal and policy structures surrounding prison rehabilitation, the oppressive nature of the austerity policy in England threatens its advanced improvement.

**Methods:**

Using grounded theory methodology, this is the first interdisciplinary qualitative study to explore the impact of macroeconomic austerity on prison health in England from the perspective of 29 international prison policymakers.

**Results:**

The far-reaching impact of austerity in England has established a regressive political system that shapes the societal attitude towards social issues, which has exacerbated the existing poor health of the prisoners. Austerity has undermined the notion of social collectivism, imposed a culture of acceptance among prison bureaucrats and the wider community, and normalised the devastating impacts of prison instability. These developments are evidenced by the increasing levels of suicide, violence, radicalisation and prison gangs among prisoners, as well as the imposition of long working hours and the high levels of absenteeism among prison staff.

**Conclusions:**

This study underscores an important and yet unarticulated phenomenon that despite being the fifth largest economy in the world, England’s poorest, marginalised and excluded population continues to bear the brunt of austerity. Reducing the prison population, using international obligations as minimum standards to protect prisoners’ right to health and providing greater resources would create a more positive and inclusive system, in line with England’s international and domestic commitments to the humane treatment of all people.

## Background

Approximately 10.4 million people are currently being held in penal institutions worldwide [1]. In England and Wales, the current rate of incarceration is 155 per 100,000 people, which is slightly higher than the global average imprisonment rate of 144 per 100,000 [1, 2]. Prisons give the state access to a population that is at particularly high risk of ill-health and uneven access to services [3, 4]. The Ottawa Charter, which states that “health is created and lived by people within the settings of their everyday life” [5], recognises this fact. The United Nations’ Sustainable Development Goals 2030 further reinforced prisoners’ right to a healthy life via nine key goals, most of which address the need to end poverty and reduce inequalities within and among countries [6]. Supportive legal frameworks—such as the Basic Principles for the Treatment of Prisoners 1990 and the binding legal jurisprudence of the European Court of Human Rights—guarantee the right to health during terms of detention.

England leads the world in terms of prison health standards [7, 8], but since the country embraced austerity in May 2010, its leadership has largely stemmed from the poor prison health conditions in other countries. Austerity has precipitated a reduction in government borrowing that requires deep cuts in public expenditure in order to rebalance the economy [9]. It reflects an embracing of neoliberalism – a policy of state restructuring processes that are organised by the logic of economic efficiency, minimal state intervention and a preference for individual rather than collective rights [10].

Although austerity is a distinct concept from neoliberalism, it has emerged as a crucial means of sustaining neoliberalism’s resilience [11, 12]. The objectives of austerity mutually reinforce those of neoliberalism, particularly in terms of reducing the role of the state and the redistribution of wealth and power [13]. Thus, the need to secure deficit reductions in the short-term and maintain confidence in the country’s financial stability in the long-term fuels the austerity agenda [14] and signposts a longer-term shift towards neoliberalism. This shift is evident in reductions across public sector spending, the framing of health and welfare as individual rather than collective duties, and the privatisation of prisons in England. These dynamics, including specifically prison spending reductions, will be critically explored in this article.

The Coalition Government has claimed that the global economy made such austerity unavoidable [15]. It frontloaded large spending cuts to public programmes, asserting that this was the way to create an acceptable equilibrium [14]. The objective was to appease the financial market, cutting public sector spending without raising taxes to meet the burgeoning deficits, so that it could continue to borrow at reasonable interest rates [14]. As a result, a pre-emptive deflation strategy was devised, similar to the conditions implemented in Greece, Ireland, and Portugal in the same period, even though Britain is not a member of the Eurozone and thus does not qualify for financial assistance by adhering to stringent austerity packages [14, 16].

Recent studies have demonstrated a regressive distributional effect that follows spending cuts, particularly impacting those who depend on public services. They illustrate the reduction in funding for preventive family support and early intervention services [17]; an increase in the mortality rate among pensioners aged 85 years and over, which has been linked to unprecedented reductions in income support [18]; and an increase in suicides and the prescription of antidepressants for patients with mental health issues [19]. In effect, these studies demonstrate the clash between efficiency, effectiveness, and equity, showing that that the burden of adjustment is not shared symmetrically, and thus it has resulted in the deepening of poverty and inequality.

Whitehall reduced the funding for HM Prison and Probation Service by 22%, from £3.48 billion in 2009/2010 to £2.71 billion in 2016/17, which led to a 30% reduction in prison staff between 2009 and 2017, even as the number of people incarcerated remained broadly consistent [20, 21]. The introduction of the Prison Unit Cost Programme in 2012, which is also known as the Benchmarking Programme, resulted in consolidated pay structures for management and operational staff, along with early retirement offers, redundancy, fixed-term contracts for the existing workforce, and the introduction of new pay levels in line with market rates, which were often lower than existing staff salaries [22]. In parallel, structural measures were undertaken, which consisted of reducing headquarters and closing small and less cost-efficient prisons [22].

Fiscal austerity has been a driving force in the reinforcement of individualism in English society. Politicians have reframed public issues, such as health, poverty, and social welfare, as individual choices rather than collective responsibilities, thereby providing the state with grounds to relinquish its moral obligations [23]. Wilkinson and Pickett [24] have linked inequality to the neutralisation of social solidarity, which leads to increasing levels of punitiveness; this is particularly relevant to the theory of imprisonment. Whilst neoliberalism assumes that society consists of rational and capable individuals who can make their own choices and decisions, the prison population is generally poor and sick, and confinement gives prisoners no alternative but to depend on state provisions during their imprisonment. Hence, the irony of austerity is enshrined in the fact that working people and the poor, not those who engineered or profited from the asset bubbles, bear the brunt of the resulting financial hardship [25].

Neoliberalism has also shifted the concept of social protection that is fundamental to the welfare state to the ideal of social risk management [26]. Diminishing trust and fading social capital resulting from individualism have accelerated what Horan describes as the abandonment and containment by government actors of society’s most marginalised populations [27]. Cohen’s implicatory denial theory intersects with Horan’s explanation, ascribing the sociology of denial theory to the notion of authorisation (whereby officials act as ordered by the state, and their duty to implement the austerity programme supersedes their moral principles, despite the resulting violation of prisoners’ entitlement to health and wellbeing provisions), routinisation (which seeks to normalise catastrophic events, such as impeding access to services and subjecting prisoners to precarious living conditions), and dehumanisation of subjects (in this case, prisoners) as undeserving members of the community [28].

A state is truly neoliberal when it utilises the market to govern the distribution of social goods and services according to the market logic of efficiency and effectiveness [29]. England’s prison privatisation suggests it meets that standard. The Prison Unit Cost Programme 2012 required public prisons to achieve rates of efficiency as high as those in the private sector, at the expense of basic standards and without reducing the prison population [30]. Research shows that privatisation of penal institutions and various in-house services heightens a sense of doubt, fear, and insecurity among the public regarding the quality of public services [31]. The lack of accountability and the inferior quality control effected by private security providers are clear [32] with growing evidence from the United States and Australia suggesting that commercial interests subordinate the role of rehabilitation in prisons [33, 34].

In 2017, reflecting the impact of seven years of austerity, inspections of English prisons carried out by both the European Committee for the Prevention of Torture and the English HM Inspectorate of Prisons documented impeded access to prison healthcare and productive activities due to the lack of discipline officers, as well as lengthy confinement within locked, overcrowded, and poorly maintained cells [20, 35]. Suicides among prisoners between June 2010 and June 2018 increased by 23% [36]. English prisons also saw an unprecedented 31,025 assaults in the 12 months to March 2018, as well as an increased presence of novel psychoactive substances, which have been associated with violence, debt, organised crime, and overdoses [35–37]. There is also reason to be concerned that high staff turnover and exposure to unhealthy, stressful, and poor working conditions inside prisons is putting staff health at risk. In 2018, HM Prison and Probation Service recorded an average loss of 9.3 working days due to staff illness, up from 9.1 days in 2017, with the suggestion that the strain of the job, and mental health and behavioural disorders, including stress, constituted 32.8% of absences [38]. By contrast, sickness absence has fallen across public sector organisations in recent years [39].

Despite these developments, the increased scholarly interest in the macroeconomic ideology of austerity has yet to articulate its impact on prison health in England. The legal and policy risks associated with the continuation of austerity and its effect on prison health, along with the mitigation strategies, require further clarification [40]. This is the first interdisciplinary qualitative study to attempt to address these scholarly gaps from the perspective of international prison policymakers.

## Methods

### Study design

Constructivist grounded theory, which builds theory from qualitative data [41], was used to examine the effects of austerity on prison health from the perspective of international prison policymakers. It is an inductive approach used in qualitative research to build theory, which is characterised by a juxtaposition of systematic and flexible guidelines for collecting and analysing data as theory construction takes place [41].

The meaning of austerity and its impact on prison health was co-constructed between the researcher and the participants, whereby the researcher stimulated in-depth discussions on how the participants made sense of austerity. The informants’ experiences, along with the context in which they occur, played a key role in precipitating the impact of austerity on the prison health agenda in England. Doing so has ensured that the theorisation process is grounded in empirical data from the participants who have experienced prison policymaking at the international level.

There were 29 participants, who were all considered to be “elite” in that they engage with policymaking activities in prison health, occupy authoritative positions in the field and have discretions to influence political outcomes more than members of the general public [42]. There is almost a complete absence of previous research concerning the elite community in the prison health field. Their participation in this research is key in highlighting their experiences of responding to the policy imperatives that resulted from the austerity regimes both on an international scale and in England. In line with purposive sampling [43], policymakers from key organisations pertinent to international prison work, such as the United Nations, the World Health Organization and the Council of Europe, along with other non-governmental organisations such as Amnesty International and the Association for the Prevention of Torture, were invited to provide accounts on the research topic.

Recruitment involved purposive sampling, theoretical sampling and snowball sampling. Purposive sampling entailed seeking out potential participants who were able to provide accounts on the topic being investigated. For theoretical sampling, additional participants were sought because they seemed likely to have perspectives that could either support or challenge the tentative findings [44]. Snowball sampling occurred because the participants allowed me to use their authority and professional contacts to access colleagues operating within the same field [45].

The inclusion criteria were as follows: rich experiences, decision-making power within the organisational hierarchy and knowledge of the prison health system in England. Details of the participants’ professional designations are provided in Table 1. On average, each participant holds two appointments, and the majority operate within the prison and health sectors. Thus, the total number in the individual groups would exceed 29 but there were only 29 individual participants.

**Table 1:** Background of participants

Participant professional designations	Number of participants
Prison	16
Health	13
Academic	11
Regulatory	8
Legal	6
Non-governmental organisations	5

### Data collection

A multifaceted recruitment plan was implemented over a 17-week period from December 2017 to April 2018. The majority of the participants were interviewed at their respective office locations in six different European cities: London, Vienna, Geneva, Amsterdam, Strasbourg, and Dublin. The interviews lasted between 27 and 96 minutes ($\overline{\mathrm{x}}$_duration_ = 54 minutes). The interview length helped to establish rapport and trust with the participants and thus to elicit more in-depth responses.

A semi-structured interview format was employed [41]. The interview guide began with an open question (“In what way did this study appeal to you?”), followed by these topics in sequence: the current state of health in English prisons, the ways in which fiscal reductions have impacted prison health in England, how policymakers have attempted to balance the mandate of austerity against the international obligations on prison health, and alternatives to austerity. The least intrusive questions preceded the more intrusive ones, whilst loaded questions, closed-ended questions, and poorly ordered questions were avoided [46]. Through the use of directed but open-ended exchanges with participants, the aim of the interviews was to elicit the participants’ own accounts of their experiences and perspectives, which are usually absent from official documents on international prison health.

Appropriate probing techniques were used to prompt further elaboration and clarification of participants’ initial responses. Lilleker’s [46] method of probing participants was used as a framework:

- “Detail-oriented probes” helped to obtain more information about the phenomena participants referenced. For instance: “We tend to focus on the impact within prisons when we discuss the impact of austerity on prison health. How about the impact outside of prisons, particularly when the majority of prisoners will eventually be released into the community?”
- “Elaboration probes” which required the participants to expand on their initial response were used. For example: “You briefly mentioned one of the impacts of austerity on prisons, which is the reduction in the number of prison officers. How is this impacting the delivery of the prison health agenda in English prisons?”
- “Contrast probes” referenced two contrasting ideas: “You mentioned that the government has obligations under the European Convention of Human Rights to protect the rights of prisoners to set healthcare provisions. Why, then, are we seeing increasing barriers to access healthcare provisions during the time of austerity?”
- “Criticism probes” involved introducing criticism of the system from previous interviews to obtain a new perspective as part of data triangulation. This included: “Some previous participants mentioned the culture of acceptance, where we simply accept austerity being imposed on prisons and other sectors, without any challenges from the policymakers and service users. How do you respond to this observation?”

A reflective journal was maintained throughout the fieldwork as an audit trail [47]. All interviews were audiotaped and transcribed verbatim. The transcripts generated more than 499 pages and 195,680 narrative texts for analysis.

### Data analysis

Using NVivo 11 software, three stages of coding were undertaken: open coding, focused coding, and axial coding [41]. First, the open coding process commenced by labelling each line of text by focusing on specific phrases. Gerunds were coded in order to capture opinions directly described by the participants, to ensure that interpretation remained close to the data [41]. These ranged from “labelling austerity as a political ideology” and “low political interest in protecting prisoners’ right to health” to “the need for more transparency in government spending plans” and “better tax regime to recover the economy”. This allowed for a careful examination of hidden assumptions in the participants’ language. Each transcript was reduced to between 16 and 33 codes.

Focused coding followed, where the differences in codes were reconciled and emerging theories were reviewed, and axial coding, where the data were reassembled to give coherence to the developing theory [41]. These codes were developed into four axial codes, which came to organise the four subsections in the Results section below: the disappearing chain of accountability, the imposition of a culture of acceptance, the normalisation of catastrophic issues arising from austerity programmes, and alternatives to austerity.

Data saturation was achieved at 29 interviews, which indicated that no new themes appeared in the interviews [41]. Frequent discussions with the research supervisory team and research collaborators helped to sense-check the data analysis to demonstrate rigour.

### Establishing trustworthiness

Specific measures related to credibility were undertaken to demonstrate the trustworthiness of the study, specifically in relation to the provisions of in-depth description of the phenomenon, multivocality, data triangulation and peer debriefing [48]. To achieve credibility, in-depth descriptions of the phenomena being examined – via concrete, rich, and varied findings from different participants from diverse backgrounds [49] – are operationalised as part of the Results section. Multiple and varied voices were showcased to demonstrate the richness of the data, with 29 participants representing 33 different organisations participating in this research.

Next, triangulation of data sources was used during the interview phase to look for similarities or dissimilarities between the viewpoints of the participants [48]. Finally, peer debriefing via conference presentations was undertaken at the Prison Health Research Symposium (University of Central Lancashire, United Kingdom, 20 June 2018) and the Fourth International Conference on Law Enforcement and Public Health 2018 (Toronto, Canada, 22 October 2018) to present the tentative findings of the study, which served as an external review of this study [50].

### Ethical approval

The Faculty Research Ethics Committee at the University of the West of England, United Kingdom, granted ethical approval in December 2017 (reference: HAS.17.11.054). Written informed consent was obtained from all participants and they were guaranteed confidentiality to promote candour.

## Results

Austerity has promulgated a regressive political system that shapes the contour of societal behaviour and preference towards social issues, which has exacerbated the existing poor health of the prisoners. This theoretical model emerged out of four data categories from the interviews with 29 international prison policymakers, as delineated below.

### Disappearing chain of accountability

The introduction and sustenance of austerity measures signalled the deliberate disappearance of the government’s chain of accountability for prison health in England in three manoeuvres. First, it justified austerity measures across the public sector, including prisons, as the only possible response to the international economic downturn. However, several informants felt that the extreme fiscal measures were grounded in ideological belief rather than credible evidence, particularly given that the economy has been showing signs of returning to prosperity (Table 2a).

**Table 2:** Disappearing chain of accountability

Theme	Illustrative quotation
2a) Challenging the legitimacy of austerity	[T] he economy's picking up now, not just in Europe but internationally, so why continue with austerity? It is fundamentally about the realignment of state provisions. (Participant 12, Advisor to a European intergovernmental human rights organisation)
2b) Framing austerity as a matter of *highpolitik*	[These measures] are framed as unavoidable … . If you present something as just a technical issue and not a political issue, there is no space for public engagement. … [A] usterity was presented in such a way. (Participant 19, Former president of a European anti-torture committee)
2c) The danger of prison privatisation	[H] ealthcare is a public good. Once you put it into a setting where it becomes dependent on a resource, you instantly create a problem where you might have unequal access to that resource. It changes the nature of the relationship between the professional and patient in ways that many professionals don’t want to happen. … [I]t's dangerous on a number of fronts. (Participant 18, Advisor to a European administration organisation)
2d) Lack of a rehabilitation culture in private prisons	The rehabilitation rates of inmates are lower in private prisons [as measured in recidivism rates]. The [quality] of services and working conditions in English prisons was worse in private prisons than in public prisons. (Participant 8, Policy lead of a European public sector trade union organisation)
2e) The state’s duty of care towards prisoners’ health	[I] t was the state that took a person’s liberty, so it is the state’s full responsibility, *direct responsibility*, to care for prisoners in a direct way and not to outsource it. (Participant 23, Advisor to a European intergovernmental human rights organisation)
2f) Prison privatisation increases long-term transaction costs for the government	At the end of the day, [a private prison is] either trying to make some kind of profit, and even if they’re not for profit, they’re looking to sustain their contract. (Participant 20, Public health specialist at an international health organisation)

Second, the government sustained the austerity measures by framing them as a matter of *highpolitik*, to limit the public’s ability to consider such measures in an informed manner (Table 2b). Bearing in mind that societal members will inevitably look after their own interests during testing times, prisoners’ health needs will not form part of the societal discourse, especially when the societal members are all vying for dwindling public resources. As such, compared to other welfare areas such as housing, education and employment, which are subject to greater scrutiny, the public have limited ability to secure governmental accountability in respect of prison health.

Third, informants attributed the disappearance of state accountability for prisoners’ health to the pernicious privatisation of the justice sector in England. All participants were unequivocally opposed to privatisation, as access to healthcare can turn into a trade commodity (Table 2c) and private prisons lack a rehabilitation culture (Table 2d). Besides asserting that prisoners’ health remains part of the state’s duty of care (Table 2e), private prisons require more hands-on management, which inevitably increases the government’s long-term transaction costs (Table 2f).

### Imposition of a culture of acceptance

Informants felt that austerity had led to a culture of acceptance among English prison policymakers. Although the participants described austerity measures in neutral terms early on in their interviews (Table 3a), most indicated that austerity did not afford any opportunities for them to manoeuvre efficiently and creatively. In fact, due to its inherent political baggage, these officials avoided using the term “austerity” (Table 3b). Instead, the participants resorted to phrases such as “lack of funding” (Participant 17, Advisor to a European intergovernmental human rights organisation) and “working under financial pressure” (Participant 4, Advisor to a European intergovernmental human rights organisation). These constraints, they explained, limit their ability to acknowledge how austerity has directly mediated the aspirations of prison health. They felt morally compromised but unable to challenge the *status quo*.

**Table 3:** Imposition of a culture of acceptance

Theme	Illustrative quotation
3a) Opportunities that arise from austerity programmes in prisons	Opportunities to think afresh, examine what we do, and [explore] different ways of working. (Participant 22, Advisor to a European intergovernmental organisation)
3b) Avoidance of the term “austerity” by the bureaucrats because of its political nature	[W] e hardly use the word austerity … but we all know we’re working in a resource-constrained environment. (Participant 29, Consultant for an international health organisation)
3c) The lack of impact of the independent inquiry mechanisms on prison operations	[In 2017], the UK Human Rights Select Committee focused on prison operations. … [T] hen there was the Public Accounts Committee [in 2018], which considered prisons and their problems. But, the question is, do they make a difference to the Treasury? And the answer seems to be no. So, the question I would ask is “Why not?” (Participant 12) We have a system where people are dying [and] we have a system where people are falling through the cracks. The problem is that … it is a prison setting. If it happened in the community, there would be a lot of disquiet and a lot of uproar in the media. Because it happens in prisons, there is not that same level of concern about it. (Participant 4)
3d) Comparison between England and the United States with regard to cuts in public sector spending	England has done it in the most complete and consistent manner. [The government has] actually cut spending across the board. In the United States, the fiscal conservatism is very selective about where it cuts. (Participant 10, Advisor to a European anti-torture organisation)

Some participants described a culture of acceptance with respect to elected officials maintaining the public’s ignorance of evidence of the detrimental impact of austerity on prisoners’ health. Despite the existence of various independent inquiry mechanisms to hold the government accountable for its treatment of prisoners, they do not appear to overturn the harmful austerity measures (Table 3c), which corroborates the existence of a regressive political system that does not acknowledge human rights aspirations.

The fact that other parallel sectors also suffer from austerity further traverses the culture of acceptance. Contrasting the selective cuts to public programmes in the United States, several interviewees critically described the English government as remarkably consistent by comparison (Table 3d). This consistency makes it harder to argue that austerity measures discriminate against prisoners, given deep cuts to housing and social care programmes, which generally have greater public support.

### Normalisation of catastrophic issues arising from austerity programmes

According to the participants, the acceptance of austerity in English prisons has normalised the catastrophic effects of austerity on prisoners’ health. The dwindling number of discipline officers, according to most participants, has contributed towards the longer waiting times to access healthcare and prisoners not being released from their cells, which has inevitably exacerbated their boredom, stress, and anxiety (Table 4a). Many participants also illustrated a visible deterioration of the living conditions in prisons (Table 4b). They described a resultant increase in violence, bullying, and drug-taking to alleviate depression and boredom, making prisons far more dangerous (Table 4c).

**Table 4:** Catastrophic issues in prisons that arise from austerity programmes

Theme	Illustrative quotation
4a) Prisoners could not be released from their cells due to the lack of prison staff	Prisons in Dubai and Abu Dhabi [have not had] one suicide since 1995 … . All landings are locked, but all cells are left unlocked, so by day everyone can mix freely with each other. But in England, we insist on separating people and doing nothing. (Participant 13, Former head of a prison inspectorate)
4b) Deterioration of living conditions in prisons	I was shown [an English prison]. I looked at the level of dirt and the level of non-upkeep of material conditions; never mind the provision of services. It was really distressing to see. I expect to see conditions like that in some of the Balkan countries I'm working in. I do not expect to see conditions like that in England. It's sending a much bigger message rather than it simply being about austerity. It's sending the message that we just don't care. (Participant 4)
4c) Insurmountable challenges in controlling prisons	[W] hen you have lower number of staff, and fewer secure prisons where it is more stressful, people start looking to themselves and other structures, informal structures, especially gangs, for their security. (Participant 25, European human rights specialist)
4d) Declining level of health and well-being among prison staff	Often with male prison officers, there is a reluctance to actually take sick leave so as not to put pressure on colleagues who would have [to do] overtime. [It is] not always easy to talk about it. In France [which has experienced similar austerity measures to England], there is a high rate of suicides among prison staff, more than in the general national population. So, on health and safety, we can clearly see the impact has been extremely harmful. (Participant 8)

Similarly, some participants highlighted that a reduction in the workforce has contributed to the rampant use of overtime among prison officers due to staff shortages. They also reported an increase in presenteeism: prison officers come to work sick because they feel they have little choice. The workforce inevitably suffers from stress, sickness, and other medical-related conditions as a result of performing within an already stressful environment (Table 4d).

As there are no signs that austerity will be reversed, numerous participants predicted soaring rates of suicides, burgeoning numbers of riots and hostage-taking incidents, an increase in recidivism, and a rise in the spread of illnesses in prison environments due to a lack of treatment. They described the current instability as the tip of the iceberg, which will only subjugate the institution if there are no improvements going forward.

### Alternatives to austerity

Every participant recommended reducing the size of the current prison population in England as a first measure. Reviewing sentencing policies, addressing the causes of soaring incarceration rates, and utilising economic arguments to support the decarceration policy would create a population suited to the current size of the prison workforce (Table 5a). The majority of the participants also advocated using international concordats as minimum standards to protect prisoners’ right to health, with intergovernmental organisations as well as international pressure groups providing greater oversight (Table 5b).

**Table 5:** Alternative measures to austerity

Theme	Illustrative quotation
5a) Calls to reduce the current prison population	[P] risons, like hospitals, are expensive. Prison health is best served when the general principle of avoiding sending people to prisons is applied … . In England, and in several other Western European countries, many people are sent to prison who should not be sent to prison. [They would] be much better looked after in society by the health and welfare system. (Participant 2, Consultant for an international health organisation)
5b) Intergovernmental organisations and pressure groups to provide greater oversight of prison health obligations	[It’s] important that you have civil society organisations and independent monitoring boards involved … seeing what’s happening in prisons, writing reports and telling the rest of the public what’s actually happening within [prisons]. (Participant 18)
5c) The need for a social conversation about taxation	We need a big social conversation about taxation. We view taxation as a dreadful imposition by the government. Actually, it’s a resource. People should be paying their taxes. Corporations should be paying their taxes. We shouldn’t be encouraging people to avoid them in any way. It’s fundamentally about deciding on the purpose of taxation and kind of reframing it, not as an imposition on individual freedom, but actually a resource for the whole of society and something we all benefit from. (Participant 29)
5d) Enticing public interest via a transparent discussion of prison spending	[E] ven if people are not interested in human rights and they are not interested in addressing the health needs of vulnerable and marginalised people, they usually care about how much money they spend. (Participant 21, Regional Lead of an international health organisation)

Finally, all participants detailed the greater resources that the English prison system requires—more prison officers, better maintenance and upkeep of facilities, and greater investment in rehabilitation—which should help to make these institutions more stable. They argued that the government would be able to afford such expenditure if it tackled tax avoidance schemes prevalent among multinational corporations and wealthy individuals (Table 5c). Such a strategy would need to be accompanied by the message that it would increase the monetary supply. Entwining current prison health spending across Europe with public discussions would ensure a more transparent discourse on prison spending (Table 5d).

## Discussion

This is the first in-depth qualitative study to explore the impact of macroeconomic austerity on prison health in England from the perspective of international policymakers. Austerity has enabled a regressive political system that shapes the contour of the societal stance towards social issues, which has as a result worsening the existing adverse health of the prisoners.

First, the disappearance of a sense of accountability for prisoners’ health has been demonstrated via the reduction in the prison budget by 22% between 2010 and 2017 [15, 20, 21]. The interviewees point out that the present situation belies the claim that a recession requires austerity. United Kingdom’s economy, the fifth largest in the world, is continually growing [51]. It has never returned to the low recession point of 2009 and growth has often over 2% since 2013 [51]. The claim that one of the largest economies in the world requires austerity in terms of government spending and pre-emptive deflation measures reveals that austerity is simply a political choice. Whilst providing further evidential support for the existing austerity studies [14, 16], considering that the impacts of austerity on prison health have yet to be theorised, this research provides much needed preliminary evidence to both politicians and policymakers of the threats to health and rehabilitation based on about a decade’s worth of data.

The increasing influence of individualism further weakens community solidarity and abandons marginalised populations [17–19], including prisoners [23, 26, 27]. The present study provides a continual thread of evidence that fiscal retrenchment has demonstrated the mismatch between efficiency and equity, which adversely affects those at the margins who are extremely dependent upon public sector support. Echoing Wilkinson and Pickett [24], this study finds that such a development increases the punitiveness inflicted on prisoners, as confirmed by the continual violence and harmful living conditions found in English prisons. Those who played no part in the economic turmoil bear the brunt of austerity [25].

The sustenance of prison privatisation parallels the diminishing state presence. Consistent with O’Hara [31], privatisation amplifies a sense of insecurity regarding the quality of public services. Studies from the United States and Australia have shown that private prisons do not rehabilitate prisoners as well as public prisons do [33, 34]. The participants in the current study cite similar oversight in private English prisons, which have not received the same research attention as their US and Australian counterparts. In providing greater insight into the fact that private entities lack accountability and deliver inferior public services [32], this research provides preliminary findings that the privatisation of prisons in England increases monitoring costs for the government in the long run and, overall, contradicts the cost-saving drive of government policy during austere times.

Second, austerity has implanted a culture of acceptance among prison bureaucrats in England. The policymakers described avoiding the term “austerity” to chronicle the reduction in prison funding in favour of words with more neutral connotations. They have no alternative but to fulfil their duty to execute austerity programmes, by which unsafe living conditions are becoming common in English prisons, supporting Cohen’s sociological theory of implicatory denial of authorisation, routinisation, and dehumanisation of subjects [28]. Such conditions violated the healthy living aspirations as guaranteed by the Ottawa Charter [5] and prisoners’ right to a healthy life referenced in the United Nations’ Sustainable Development Goals 2030 [6]. They also threaten to raise recidivism and contribute to a less safe society, and expose the state to legal liabilities for violating prisoners’ right to health.

Furthermore, this study illuminates a phenomenon that existing research had not articulated, whereby the culture of acceptance also prevents the independent inquiries from overturning the detrimental impact of austerity on prisoners. The study participants negatively compare the blanket imposition of austerity measures in England to the public sector cuts in the United States, asserting that it contradicts England’s view of itself as a welfare state.

Third, while the persistence of austerity normalises the increasing levels of suicide and violence [35–37], the present findings provide a wake-up call regarding the cumulative level of disengagement among the prison population which breeds radicalisation and pockets of prison gangs. Institutional instability makes the existing workforce susceptible to high levels of stress, as evidenced by the increasing absence rate due to sickness even as other public sector organisations have witnessed declining levels of sickness absence [38, 39]. Moreover, this research highlights a worrying trend whereby the existing workforce experiences long working hours and high levels of absenteeism, which could attract legal culpability. In his Budget Speech of October 2018, the UK Chancellor of the Exchequer Philip Hammond declared that “the era of austerity [is] finally coming to an end”, but despite this, he had also insisted that financial discipline would remain in place [52]. This indicates that the austerity era may in fact continue and that real changes have yet to emerge. As such, suicides, violent incidents, reoffending and the spread of communicable diseases will only continue to increase, thereby heightening the risk of inflicting torturous, inhumane and degrading treatment on both prisoners and prison staff.

All participants called for a reduction in the current prison population in England, which would allow prisons to concentrate on a smaller population cohort [1]. Based on the premise that a lack of financial resources should not arbitrarily interfere with prisoners’ enjoyment of good health [5], intergovernmental and international non-governmental organisations should monitor, scrutinise, advocate, and publicise the level of compliance in every country under their jurisdiction, England included, in terms of the established international standards of health. Finally, tackling tax avoidance issues among multinational corporations and wealthy individuals, as well as publishing the details of prison spending for transparent comparison against other nations, could have an impact. These suggestions could be considered to reverse the structural deterioration of prisons and they may be used to improve the rehabilitation of prisoners. As imprisonment and austerity are operationalised as a political choice, systemic reformation will largely depend on political courage.

Although this research contextualises the impact of austerity on one of the most marginalised segments of society, its unique contribution may be qualified by the fact that the sample comprised 29 international prison policymakers. This exclusivity therefore precludes the views of prison governors and staff, and the prisoners themselves, a limitation that should be addressed in future research. Nevertheless, the findings herein have great relevance to countries beyond England, including all those that adopted austerity in relation to prisons following the 2008 global economic recession. Further studies might investigate the effects of austerity on those countries’ prison systems and build on the assertions illustrated within this study.

## Conclusions

This study indicates that the austerity measures introduced by the English government have pervasively affected the health of prisoners and prison staff. The guiding norms of austerity organise the current social order, which affects societal treatment of prisoners, who are disadvantaged and marginalised. The far-reaching impacts of austerity as a political option have been illustrated by undermining the notion of social collectivism, imposition of a culture of acceptance among prison bureaucrats and the wider community, and normalisation of the devastating impacts of prison instability.

The prolonged implementation of the austerity regime will only exacerbate the deepening health inequalities in prisons, cause prisons to reach a breaking point, and, finally, increase the likelihood that England will be legally accountable for breaching international and European human rights principles regarding humane treatment during detention. In this regard, this paper provides alternatives to austerity, which include reducing the prison population, using international concordats to protect prisoners’ entitlement to health and healthy living, and providing more resources to English prisons, thereby safeguarding the aspiration for prison rehabilitation, albeit requiring fundamental shifts in the political paradigm.

Nearly a decade has passed since the introduction of the political rhetoric of austerity in England. Real changes have not dovetailed with the recent government announcement proclaiming that austerity is coming to an end. Bearing in mind that both austerity and imprisonment are in fact political choices, more research is required to increase our appreciation of the arbitrary use of these policies in a contemporary, rights-based society. This research offers a starting point that provides a narrative calling for fundamental reform to a harmful system in order to create a more positive and inclusive system, in line with England’s international and domestic commitments to the humane treatment of all people.

## Data Availability

Due to privacy restrictions, the datasets generated and analysed in the present study are not publicly available, as they may contain information that could compromise the participants’ privacy. For the participants, reputational risks could outweigh the need to make the datasets available on the publisher’s website.
